# Contexts of care for people with differences of sex development: Diversity is still missing in the laboratory routine

**DOI:** 10.1515/medgen-2023-2037

**Published:** 2023-08-16

**Authors:** Alexandra E. Kulle, Martina Jürgensen, Ulla Döhnert, Lisa Malich, Louise Marshall, Olaf Hiort

**Affiliations:** Campus Kiel/Christian-Albrechts University of Kiel Division of Pediatric Endocrinology and Diabetes, Department of children and adolescent medicine I, University Hospital of Schleswig-Holstein Rosalind-Franklin-Str 9 24105 Kiel Germany; Campus Lübeck/University of Lübeck Division of Pediatric Endocrinology and Diabetes, Department of Pediatrics, University-Hospital of Schleswig-Holstein Lübeck Germany; Campus Lübeck/University of Lübeck Division of Pediatric Endocrinology and Diabetes, Department of Pediatrics, University-Hospital of Schleswig-Holstein Lübeck Germany; University of Lübeck Institute for the History of Medicine and Science Studies Lübeck Germany; Campus Lübeck/University of Lübeck Division of Pediatric Endocrinology and Diabetes, Department of Pediatrics, University-Hospital of Schleswig-Holstein Lübeck Germany; Campus Lübeck/University of Lübeck Division of Pediatric Endocrinology and Diabetes, Department of Pediatrics, University-Hospital of Schleswig-Holstein Lübeck Germany

## Abstract

The 2006 Chicago consensus statement of management of disorders/difference of sex development (DSD) has achieved advantages in clinical care and diagnosis for patients and families affect by DSD. This article provides a brief overview of contexts of care for physicians, and points out specific challenges in clinical practice that have arisen from the transformations of the sex/gender system in recent years. We focus on the impact of diagnosis and laboratory measurements. Both laboratory measurements and hormonal therapies still depend on the binary system. One problem is the lack of reference intervals for the different forms of DSD, which means that diversity is often neglected. In the following, we will give a brief insight into this complex topic.

## Introduction

**Figure 1:**
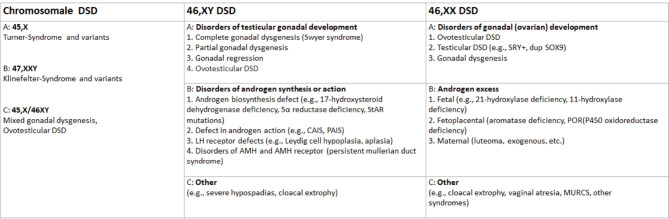
The nomenclature for classifying DSD, adapted from Hughes et al., 2006 [1],DSD, disorder/differences of sex development, StAR steroid hormone acute regulatory protein, CAIS, complete androgen insensitivity syndrome, PAIS partial androgen insensitivity syndrome, AMH, Anti-Müller-Hormon, POR, p450 oxidoreductase, MURCS, Müllerian duct aplasia–renal agenesis–cervicothoracic somite dysplasia

DSD comprise a heterogenous group of congenital conditions affecting chromosomal, gonadal and anatomic sex [1]. They are classified according to chromosomal sex into 46,XY DSD, 46,XX DSD and chromosomal DSD [1]. Each main group encompasses several subgroups oriented towards a specific clinical or molecular diagnosis [2] as shown in figure 1. There is a wide spectrum of overlapping phenotypes and an equally wide spectrum of aetiologies [3, 4]. With an estimated incidence of 2 in 10,000 live births [5], DSD are rare conditions, with some enzyme deficiencies being ultra-rare (e. g. P450scc deficiency only described in single cases). The conditions themselves do not require immediate medical intervention unless there is concomitant adrenal insufficiency or severe malformation. Nevertheless, interdisciplinary medical and psychosocial care is needed for establishment of a precise diagnosis, comprehensive education of patients and relatives, and lifelong holistic care and support. This is provided at centres of expertise, with multidisciplinary teams following national and international consensus guidelines [1, 6, 7]. In this article, we outline collaborative efforts to improve care for people with DSD and highlight lingering challenges of diagnosis and management in the sociocultural context.

## Nomenclature and the sociocultural context

Diagnostic categories such as DSD do not arise exclusively within medicine, but always in exchange with social practices and cultural discourses. In DSD, this is particularly challenging, as they concern sex development and expression, which are a central feature of a person’s self-understanding and have both social and legal significance. In Western societies, notions and roles of sex, gender and sexuality have changed significantly in recent decades, with implications for the management of DSD. For a long time, societies have been shaped by a binary sex/gender model of male-female, which – with the strong involvement of medicine – has led to the stigmatisation and pathologisation of individuals who did not clearly fit into binary schemes. Since the 1990s, patients and activists have protested against the exclusivity of the binary model and the pathologisation of “intersex” variants in medicine. In part, these protests have led to a dispute between patient representatives and medical professionals that has gradually changed the care of people with DSD since the early 2000s [8]. This has included a change in nomenclature. Older and colloquial terms such as intersexuality, pseudohermaphroditism and hermaphroditism have been contested because they can be confusing, and some patients perceive them as potentially derogatory. In addition, these old terms were too vaguely defined from a medical point of view and thus harboured the risk of information being lost (e. g. in the exchange between physicians). The 2006 “Consensus statement on management of intersex disorders” [1] therefore proposed the now common umbrella term “DSD”, which at the time stood for “disorders of sex development”. In the years following the 2006 publication, there has been a renewed discussion and reflection on nomenclature. The two-sex model has also been questioned and criticised in parts of the humanities, gender studies and history of science because it excludes diversity [9–11]. Activists also criticised the term “disorder”, which despite better intentions perpetuates old tradition of pathologisation. As a result, some more recent medical publications now use the acronym DSD to mean “differences of sex development” (instead of “disorders”). These changes in nomenclature highlight the complex situation of DSD, where conflicting movements and debates collide. The two-sex model is still considered the norm in everyday situations; some manifestations of DSD are also associated with “disorders” that require medical treatment. But people with DSD increasingly live in a more inclusive sociocultural context in which their diversity is recognised.

## Diagnosis of DSD

**Figure 2:**
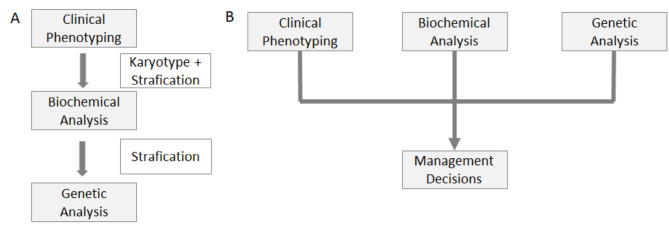
Diagnostic approaches to differences/disorders of sex development (DSD), adapted from Audi et al., 2018 [14],. (A) the traditional pathway approaches the diagnosis in a stepwise stratification. In particular, targeted genetic test are often only performed after biochemical guidance. (B) The recommended multidisciplinary approach in which the information on clinical phenotyping is considered in parallel with the biochemical (hormonal) data and genetic results (the karyotype and the candidate gene results) in an integrative manner.

Establishing a diagnosis for patients with DSD remains one of the most difficult tasks in medicine [12], but an accurate diagnosis is crucial to estimate comorbidities and associated risks for variant-carrying family members. Traditionally, DSD diagnoses have been confirmed by a stepwise stratification starting with clinical phenotyping and karyotyping, followed by biochemical and genetic testing [1]. 46,XX DSD caused by 21-hydroxylase deficiency [13] is an exception since it is part of the newborn screening in many countries due to the potential for life-threatening salt-wasting crises. For all other forms of 46,XX and 46,XY DSD, genetic approaches have made significant progress in recent years, including next-generation, whole exome, and whole genome sequencing [12, 14]. The latest approach to diagnosing DSD has been described detailed by Audi et al. [14], as shown in figure 2. Briefly, the initial step is to determine the karyotype to define the DSD subgroup, chromosomal sex DSD, 46,XX or 46,XY. Second, a copy number variation should be done. A steroid analysis is recommended for all neonates and young infants to detect potentially life-threatening acute adrenal insufficiency. A multidisciplinary team is recommended for all issues, as physical phenotyping and hormonal evaluation should be done in parallel with the genetic study. Next-generation or whole exome sequencing is preferable for analysing candidate genes if there is no suspicion of a monogenic DSD cause. Whole genome sequencing is currently reserved for the characterisation of novel DSD genes and is not yet recommended in clinical practice. Diagnosing DSD requires always a multidisciplinary team [14].

Biochemical diagnosis has also changed in the last decade, and mass spectrometry-based assays are now used for hormone determination. These methods enable steroid hormone analysis of whole profiles from one sample, including all the hormones needed for diagnosis of DSD. This includes targeted approaches using liquid chromatography tandem mass spectrometry as well as untargeted approaches using gas chromatography mass spectrometry [15].

## Impact of medical diagnosis in DSD 

It often takes years to establish a definite diagnosis in patients with rare conditions. Many people [16] with a DSD receive no genetic diagnosis at all [12]. This has an impact on several aspects of dealing with the condition – both for the affected individuals and their families and for the medical staff. In general, the lack of a precise diagnosis causes uncertainty that patients and families usually perceive as a great burden [17–19]. This uncertainty relates to questions about the cause of the condition, the prognosis with regard to physical and psychological development, possible comorbidities and – last but not least – therapeutic options. Without a diagnosis, the condition can hardly be understood and hardly be handled. As Ilgen et al. [20] have pointed out, diagnosis is “*a way of making meaning of the situation in order to better understand and manage a problem.”* The diagnosis is often perceived as a relief – but on the other hand, receiving a diagnosis also means receiving a label, which may also cause sorrow, disappointment, hopelessness or shock [17, 21]. This is particularly true when the diagnosis indicates that there is no “cure” or that there are only limited therapeutic options, as is usually the case with DSD.

Negative effects of a diagnostic label per se can be observed on both a psychological and a societal level. In addition, diagnostic labels usually have an impact on self-identity and social identity, and can be associated with social stigma [22]. The labelling in the context of a medical diagnosis *“is the recognition that a person with a particular diagnosis differs from the norm in ways that have social significance*” [23]. Since the diagnosis of a form of DSD touches on highly sensitive areas of one’s individual and social life (e. g. self-image, gender identity, sexuality, reproduction, gender roles, social norms and values), the assignment of a DSD diagnosis has a blatant impact on the affected individual, the family and the social environment. This may explain why many people who are assigned to the DSD spectrum because of their diagnosis vehemently reject this label – this particularly applies to people with CAH, Turner or Klinefelter Syndrome. In summary, good diagnostics are essential for good medical care and also for processing and constructively dealing with the situation of the individuals affected. Bearing in mind that the diagnosis can also have negative effects on those affected adequate medical and psychosocial care for individuals with DSD and their families has to be provided – not only at the time of diagnosis, but over an entire lifespan. 

## Recommendations and Activities 

The international and national guidelines mentioned above recommend interdisciplinary medical and psychosocial care for DSD at specialised centres, thorough education of patients and relatives, and shared-decision making with regard to all therapeutic options [1, 6, 7]. While the Chicago consensus statement still considered early gender assignment important [1], the German Ethics Council has argued against irreversible therapies to achieve gender assignment, and amongst other things has promoted the participation of children in their own therapy, and has recommended amending the German Civil Status Act to allow a third category for sex (German Ethics Council 2012) [24]. This was implemented by the legislator in 2018 with the category “diverse”. The ethical recommendations have found their way into recent guidelines [7]. The project “DSDCare” has been funded over the past three years by the German Ministry of Health to implement these guidelines and evaluate care at ten specialised centres in Germany. 

Another consequence was standardising the care of individuals with DSD by collecting and sharing data between countries, e. g. in the international I-DSD network [25], initiated in Europe. This supported further activities such as the EU COST Action DSDnet and the FP7 project DSD-Life. These collaborative activities also aimed to improve quality of care [26], as continued in the European Reference Networks (ERN) [27]. Further activities for the care of patients with DSD have previously been described by Jürgensen et al. [28].

## Challenges 

Individuals with DSD experience the same difficulties in receiving adequate care as other people with rare or very rare conditions: a need for specialised and individualised medical and psychosocial care, the presence of only a few experts, especially in adult care, long journeys to centres of expertise, and little evidence of therapies or outcomes. Below, we highlight special challenges in the diagnosis and management of DSD.

A part of diagnosis, as well as a huge part of follow-up treatment of DSD, is based on laboratory testing. Nowadays, laboratory testing has become an indispensable tool in clinical decision-making and monitoring of diseases. Nearly 70 % of physicians’ medical decisions are based on information provided by laboratory test reports [29]. Interpretation of hormone measurements requires reference intervals (RI), which include variables such as sex, age, Tanner stages, menstrual cycle etc. [30]. So far, most available RI are divided into two sexes, female and male. However, recent data show that DSD patients cannot be clearly categorised in this binary system [31]. Since December 2018 the German Civil Status Act has allowed people with DSD to use the third category “diverse” instead of male and female [24]. This option has led to substantial changes in the laboratory routine, and also changed the requisition slips, which now specify “diverse”, female and male [32]. The practical implication was not considered, since RI for “diverse” persons are now needed for medical interpretation. The laboratory information system (LIS) has to allocate patients to one sex automatically. If “diverse” is not defined and has its own RIs, the “default-value male” is used. Currently, the classification into female or male leads to wrong interpretation of values, which requires DSD experts for medical decisions based on laboratory results [6]. Interpretation of laboratory values is furthermore complicated by hormone therapy with oestrogens or testosterone.

The establishment of an RI for two sexes was already a complex and time-consuming process, especially for children [33]. For the direct method, a reference population has to be defined, consenting volunteers have to be recruited and blood samples must be taken. A minimum of 120 data points is recommended for each category such as sex or age or Tanner stage [34]. The determination of RIs for “diverse” persons using direct methods therefore seems impossible, since there are so many different forms of DSD [35]. In recent years, indirect methods using existing data such as databases and/or LIS have been established [36]. The premise for the indirect methods is that the values of most analyses result in the expected RIs and that there are usually many non-pathological values. Since all values are used, pathological results are also included, and this could influence the RI [36]. Diagnosis of DSD can only be achieved if different countries collect and share these data within the networks mentioned above. This requires complete clinical data, including medication. 

Similar challenges arise with hormone therapy for DSD, as required with impaired gonadal sex steroid production or after gonadectomy to induce puberty, maintain bone health and support physical and social well-being [37]. Studies are available for only a few diagnoses and recommendations have traditionally been based on male or female gender assignment [38]. Sex steroid profiles of individuals with DSD do not always follow the usual male/female pattern as shown in CAIS by Döhnert et al. 2015 [39]. In a subsequent study, treatment with estradiol was compared to testosterone treatment in 26 patients with CAIS and showed no significant differences in outcome and safety [40, 41], which supports individual treatment according to patients’ needs. However, with little evidence available, treatment options have to be thoroughly discussed with patients and close supervision of treatment and multidisciplinary care are recommended.

## Conclusion

This article provides a brief overview of contexts of care for physicians, and identifies specific challenges in clinical practice that have arisen from the transformations of the sex/gender system in recent years and that still need resolution. As we have shown, recent cultural, legal and ethical changes can bring different contexts of care into conflict with each other. This is especially the case with the new legal category of “diverse”, which does not fit the established infrastructures of the laboratory and therapeutic routines that continue to follow older binary logics. To improve care for people with DSD and to better address sex diversity in medicine overall, we need new research efforts. These require inter- and transdisciplinary collaborations that consider sociocultural and ethical issues as well as addressing biomedical, hormonal and genetic questions. In particular, future research programmes should lead to reflecting on the implications of nomenclature and diagnoses, modernising laboratory routines, defining appropriate RIs, and establishing innovative therapies that are precisely tailored to the concerns of people with DSD. 
